# The Parents of Prisoners

**Published:** 1895-05-25

**Authors:** 


### Modern Sociology.

### THE PARENTS OF PRISONERS.

Are we not inclined to carry the heredity theory too far? It is a law with variations—witness the mental and physical differences to be found in all families. Beyond these wholly, or almost wholly inexplicable variations, there are others wrought by education and environment which are quite as important, and it is the forgetting of this side of the question which makes heredity an excuse for fatalism, the refuge of the self-indulgent and the cowardly, and the shadow on the soul of the man who aspires to reach nobler things on a ladder made of the dead selves of himself and his ancestors. Heredity is one of the factors in life's progress, but by no means the only one, and often enough not the chief one. "Man is man, and master of his fate;" he is not the mere repetition of either the good or evil tendencies of his ancestors.

A great deal has been written and said, especially about the ancestry of criminals. The Jukes family, in particular, has attained a world-wide renown, which none of its members could have expected, as being the most perfect example known of a history of crime going on through generations. But this continual dwelling on heredity has caused a general neglect of another factor in the production of criminals, at once supplementary to heredity and counterbalancing it, namely, environment. To decide which has the greater power in leading to crime, and to what extent the will to do right may counterbalance both, demands an inquiry too far-reaching for the space at our command; but some suggestive statistics may be gleaned from the records of Elmira Reformatory, where, more perhaps than in any other prison in the world, the natural bias and antecedents of the prisoner are studied with a view to his reformation.* From these it appears that the parents of a large majority of the youths sent there are neither better nor worse than those of the average multitude who never fall into crime.

The fate, not only of the drunkard, but of the drunkard's child has been depicted in the gloomiest colours on every temperance platform in the world. Yet among the inmates of Elmira Reformatory, while those in whose parents drunkenness has been clearly traced numbered 37 per cent. of the whole, the children of temperate parents were over 50 per cent. Twelve per cent. came of ancestors whose temperance record was doubtful, and these may be credited to the drunkards. That still leaves it a clear matter of fact that the children of drunkards are not more liable to fall into crime than those of temperate parents. A still smaller proportion of the inmates can lay the onus of their criminal tendencies on the mental weakness of their fathers. In just 12 per cent. of the inmates could epilepsy or insanity be traced in their ancestors. If we are to take away, or even minimise, the personal responsibility of the criminal, we must seek for some other cause than this.

It seems that the parents of the large majority of prisoners were labourers and artisans, who had received an ordinary common school education. A considerable proportion could, however, only read and write, while not more than 5 per cent. could boast of education beyond the modest attainments of the common school. It seems, indeed, that the educated classes do not breed criminals. The professions contributed only 2 per cent. to the inmates of Elmira, though we own with regret that of these the children of doctors numbered one-third. Nor, at the other extreme, were there many inmates who sprang from pauper parents. This is in one sense comprehensible. The pauper, like the unjust steward, cannot, or will not, dig; but, unlike him, he does not add, "to beg I am ashamed." The man who will beg has less temptation to steal than another. What reason can be given, then, why these lads, the majority of whom are the offspring of artisans of moderate education, should fall into crime? In this age of high ideals it may seem sordid to suggest that a lack of thrift had anything to do with the family's decline and fall, yet in the statistics of Elmira is it written that of the 6,639 inmates whose family history is given, 79 per cent. had parents who had "no accumulations." The thing is comprehensible enough. The luxuries of the thrifty are the necessaries of the self-indulgent, and when they are lacking theft of some sort is the only resource. In the upper classes theft takes the form of running up bills without having the means to pay them; among artisans this method cannot often be practised, and the result is the form of theft, which leads not to the Bankruptcy Court, but to the gaol. For of the 6,639 inmates concerning whom (out of a total of 6,641) information could be attained, over 93 per cent. found their way to the reformatory through offences against property, and over 57 per cent. of them were between 16 and 20 years of age, when home influences were still important. It is likely, indeed, that all these youths were living at home at the time they were guilty of the crime that sent them to the reformatory, for the statistics show an even larger proportion—58 per cent.—who were living at home up to the date of their crime.

But what, then, was the nature of the home? This is the final—we think the crucial—question. The answer from Elmira is: "Positively bad, 3,257, or 49 per cent.; fair (only) 2,695, or 40·6 per cent."—89 per cent. in all. In scientific language—heredity is much, but environment is more. These lads, whose parents were not criminal, nor, often, even drunken, but were thoughtless and self-indulgent, who held up no ideal, showed no example to their children, fell easily into crime. It is noteworthy that the proportion of illiterates and imperfectly educated is much greater among the prisoners themselves than among their parents. These fathers and mothers had not striven to give their children even such advantages as they had themselves enjoyed. The home was bad, and therefore, not through an inevitable fate, not through a merciless heredity, but through the simple failure in duty of those who, having brought them into the world, were bound to guide them as well as they could through its devious mazes, these youths became numbered among the criminal classes.

*See the Nineteenth Year Book of the New York State Reformatory at Elmira.

